# P03-024 – Early onset IBD treated by tocilizumab

**DOI:** 10.1186/1546-0096-11-S1-A222

**Published:** 2013-11-08

**Authors:** R Helbling, A Nyddeger, F Angelini, A Von Scheven Gete, M Hofer

**Affiliations:** 1 Gastroenterology Unit, Department of Pediatrics, Centre Hospitalier Universitaire Vaudois (CHUV), Lausanne, Switzerland; 2 Immunology, Allergology and Rheumatology Unit, Department of Pediatrics, Centre Hospitalier Universitaire Vaudois (CHUV), Lausanne, Switzerland

## Introduction

Inflammatory bowel diseases (IBD) normally manifest in adolescent and young adults; nevertheless, in about 1% of the cases, disease onset occurs before the 1^st^ year of age. The clinical picture is often characterized by an indeterminate colitis whose clinical remission is difficult to achieve. Current treatment for IBD consists of either immunosuppressant or biological (Infliximab, Adalimumab) agents.

## Case report

A girl born from non-consanguineous parents of Swiss, Spanish and Korean origin, presented, at the age of 5 months, with bloody diarrhea, anal tags, vomiting, anorexia, mimicking a severe proctocolitis due to cow’s milk protein allergy, non-responsive to elimination diet. Infections, celiac disease, cystic fibrosis were also excluded. Immunogical investigations, NBT, ANA, ASCA and ANCA were negative, and immunoglobulins were within the normal range.Due to persistent symptoms and failure to thrive, upper and lower endoscopiy was performed showing an ulcerative ileo-pancolitis. The histological analysis confirmed a focally erosive, non-granulomatous ileo-colitis Leading to a differential diagnosis of Crohn’s disease versus indeterminate colitis. The patient clinically improvend with steroids and azathioprine. The follow-up was marked by arthritis of the ankle and knee, and erythema nodosum. She did not present severe or recurrent infections, besides those commonly associated with immunosuppresive therapy. Digestive symptoms were first cortico-dependent and eventually cortico-resistent, therefore, in alternative to azathioprine, infliximab, adalimumab and methotrexate were sequencially introduced. Despite these multiple therapies, a sustained remission could not be achieved. On the basis of the important systemic inflammation associated to joint involvement, the patient was started on tocilizumab, an anti-IL-6 receptor known for its efficacy in sytemic-onset juvenile idiopathic arthritis. The new treatment was well tolerated and induced a substantial improvement of the digestive and articular symptoms, together with the laboratory parameters, over 6 months follow up.

## Discussion

We report the efficacy of anti-IL-6R therapy in a child affected by a severe early onset IBD, not responding to traditional therapy. Recent works indicate a role of IL-10 pathway in the pathogenesis of early onset colitis. In our patient further immunological and genetical investigations are ongoing.

## Disclosure of interest

None declared

## References

[B1] Kotlarz D Loss of interleukin-10 signaling and infantile inflammatory bowel disease: implications for diagnosis and therapy Gastroenterology 2012 11 2 347 55 22549091 10.1053/j.gastro.2012.04.045

[B2] Smolen JS Consensus statement on blocking the effects of interleukin-6 and in particular by interleukin-6 receptor inhibition in rheumatoid arthritis and other inflammatory conditions Ann Rheum Dis 2013 11 4 482 92 23172750 10.1136/annrheumdis-2012-202469 PMC3595138

